# Evolving Clinical Manifestations and Outcomes in COVID-19 Patients: A Comparative Analysis of SARS-CoV-2 Variant Waves in a Romanian Hospital Setting

**DOI:** 10.3390/pathogens12121453

**Published:** 2023-12-14

**Authors:** Violeta Briciu, Daniel-Corneliu Leucuta, Monica Muntean, Amanda Radulescu, Cristina Cismaru, Adriana Topan, Lucia Herbel, Melinda Horvat, Mihai Calin, Roxana Dobrota, Mihaela Lupse

**Affiliations:** 1 Department of Infectious Diseases and Epidemiology, Iuliu Hatieganu University of Medicine and Pharmacy, 400348 Cluj-Napoca, Romania; briciu.tincuta@umfcluj.ro (V.B.); monica.muntean@umfcluj.ro (M.M.); aradulescu@umfcluj.ro (A.R.); cristina.cismaru@umfcluj.ro (C.C.); topan.adriana@umfcluj.ro (A.T.); melinda.horvat@umfcluj.ro (M.H.); mihaela.lupse@yahoo.com (M.L.); 2 The Clinical Hospital of Infectious Diseases, 400348 Cluj-Napoca, Romania; herbelucia@yahoo.com (L.H.); mihai_calinn@yahoo.com (M.C.); roxidobrota@gmail.com (R.D.); 3 Department of Medical Informatics and Biostatistics, Iuliu Hatieganu University of Medicine and Pharmacy, 400349 Cluj-Napoca, Romania

**Keywords:** SARS-CoV-2 variants of concern, COVID-19 wave, comorbidities, COVID-19 complications, vaccination status, in-hospital mortality

## Abstract

The aim of this study was to evaluate differences in the clinical manifestations and outcomes in hospitalized patients with COVID-19 in a single Romanian center during four pandemic waves determined by different SARS-CoV-2 variants of concern (VOCs). A retrospective study on 9049 consecutive hospitalized adult patients was performed between 27 February 2020 and 31 March 2023. The study interval was divided into waves based on national data on SARS-CoV-2 VOCs’ circulation. Multivariate logistic regression models were built, predicting death and complications as functions of comorbidities, therapy, wave, severity form, and vaccination status, and adjusted for ages ≥65 years. Pulmonary (pneumothorax/pneumomediastinum, pulmonary embolism) and extrapulmonary complications (liver injury, acute kidney injury, ischemic/hemorrhagic stroke, myocardial infarction, and gastrointestinal bleeding) were present, more frequently in ICU hospitalized patients and with differences between waves. The highest in-hospital mortality was found in patients presenting pneumothorax/pneumomediastinum. All of the evaluated risk factors were significantly associated with death, except for obesity and the Omicron wave. Our study highlights the changing nature of COVID-19 and acknowledges the impacts of viral mutations on disease outcomes. For all four waves, COVID-19 was a severe disease with a high risk of poor outcomes.

## 1. Introduction

The pulmonary manifestations of COVID-19 are well recognized, but COVID-19 is associated with deleterious effects on many other organ systems. The liver is the second most common organ affected after the lung [1]. While it is now widely established that acute kidney injury (AKI) is an important complication of SARS-CoV-2 infection, there is marked variability in its reported incidence. Renal injury involves triggers like renal hypoperfusion-related acute tubular necrosis, dysregulated inflammatory response, microcirculatory dysfunction, and direct viral injury [2]. Neurological complications that range from mild to severe (such as seizures, intracerebral hemorrhage, and stroke) are present in COVID-19 patients. Patients may present with stroke as the first symptom of COVID-19, while stroke is also described in COVID-19-positive patients with no known heart conditions or other underlying illnesses such as hypertension or diabetes [3]. As myocardial infarction (MI) can occur in the setting of acute stressors such as infection, hypoxemia, anemia, hypotension/shock, acute kidney injury, and congestive HF, it is unsurprising that COVID-19 patients are frequently diagnosed with MI [4]. 

The COVID-19 pandemic put unpreceded pressure on the healthcare system in Romania, as it did around the world. According to official data from the WHO, Romania reported 3,501,193 cumulative reported cases and 354.86 deaths per 100,000 people [5]. At the end of October 2021, during the Delta wave, Romania ranked in the first position globally in daily COVID-19 deaths/million people [6]. Although vaccination started in Romania in at the beginning of January 2021, the Romanian population was very reluctant towards COVID-19 vaccination [7]. According to the last available European Center for Disease Control (ECDC) update on vaccine uptake in the European Union (EU), as of the beginning of October 2023, Romania holds the second to last position from EU countries, with a cumulative uptake of 42.2% of the primary vaccination at the population level and 9.2% of the first booster, while at the European level, there is a cumulative uptake of 73% of the primary vaccination and 54.8% of the first booster [8].

With multiple factors changing during the pandemic at the global and regional levels, like therapies, vaccination, and the occurrence of new SARS-CoV-2 VOCs, we aimed to investigate the clinical course and outcomes of hospitalized COVID-19 patients in a Romanian infectious diseases hospital over three-year intervals and four pandemic waves. Our study addressed the following objectives: to investigate the differential impacts of various SARS-CoV-2 variants on hepatic, cardiovascular, nervous, and renal systems, and to identify predictive factors for mortality and major complications in COVID-19 patients.

## 2. Materials and Methods

### 2.1. Study Design and Setting

A retrospective study on consecutive hospitalized patients was performed, starting with the first hospitalized case of COVID-19 (27 February 2020) until 31 March 2023 in the Clinical Hospital of Infectious Diseases, Cluj-Napoca, Romania, which is a first-line hospital dedicated exclusively to COVID-19 patients during the pandemic. 

### 2.2. Participants

Inclusion criteria were the following: COVID-19 diagnosis (positive SARS-CoV-2 molecular diagnostic or rapid antigen test) and age ≥ 18 years old. For SARS-CoV-2 molecular diagnostic, we used an automated diagnostic tool, the NeuMoDxTM288 Molecluar System (QIAGEN, Hilden, Germany), with specific test strips, targeting the N and Nsp2 genes. The results were validated according to the producer’s instructions: negative for amplification of just the internal control, and positive for amplification of one or both viral genes. Rapid antigen tests used during the study interval included the following: STANDARD Q COVID-19 Ag Test (SD Biosensor Inc., Yeongtong-gu, Republic of Korea), STANDARD F COVID-19 Ag FIA (SD Biosensor Inc., Yeongtong-gu, Republic of Korea), Panbio COVID-19 Ag Rapid Test (Abbott Rapid Diagnostics, Lake Forest, IL, USA), and Test rapid COVID-19 Antigen (DDS Diagnostic, Bucharest, Romania).

### 2.3. Variables and Measurements

Data collected included age, gender, admittance date, duration of hospitalization, comorbidities, and vaccination status. COVID-19 pulmonary complications (pulmonary thromboembolism, pneumothorax/pneumomediastinum) and extrapulmonary complications (liver injury, AKI, MI, ischemic/hemorrhagic stroke, digestive hemorrhage, other hemorrhage, and thrombosis) were recorded, as well as medication, ICU admission, and clinical outcome. The severity of COVID-19 was defined according to the first World Health Organization classification [9] and adopted by a Romania Health Ministry Order on COVID-19 management. Disease severity was established at the end of hospitalization.

**Figure 1:**
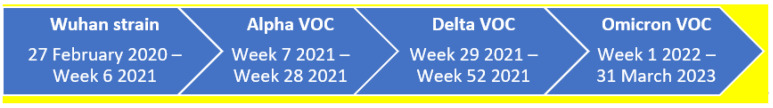
SARS-CoV-2 VOCs study intervals.

The study interval was divided, based on national data on the circulation of SARS-CoV-2 VOCs [10], into the first wave (ancestral Wuhan strain), which was the period of 27 February 2020–Week 6 2021; the second wave (Alpha VOC), which was the period of Week 7 2021–Week 28 2021; the third wave (Delta VOC), which was the period Week 29 2021–week 52 2021; and the fourth wave (Omicron VOC) (week 1 2022–31 March 2023) (Figure 1)

### 2.4. Statistical Analysis

Categorical data were presented as counts and percentages. Non-normally distributed quantitative data were presented as median and interquartile range. Comparisons between two independent groups concerning categorical data were performed with chi-squared test or Fisher exact tests (in cases where the expected frequencies were below 5 in more than 20% of the expected cells and when any expected frequency was less than 1). Comparisons between multiple independent groups concerning non-normal quantitative data were performed with the Kruskal–Wallis test. Multivariate logistic regression models were built, predicting death and complications as functions of comorbidities, therapy, wave, severity form, and vaccination status and adjusted for age ≥65 years. The assumption of multicollinearity was checked with the variance inflation factor. To evaluate the impact of sample size on our regression estimators, we calculated bias-corrected and accelerated (BCa) 95% confidence intervals using 1000 bootstrapped samples, in conjunction with confidence intervals derived from the likelihood-ratio statistic. Subsequently, we conducted 10-fold cross-validation, repeated 100 times, to further validate our model’s performance. Statistical significance was set at *p* < 0.05 for all analyses. R software 4.1.2. was employed [11].

## 3. Results

A total of 9049 patients were included. The demographic data, comorbidities, vaccination statuses, COVID-19 severity forms, therapies, outcomes, and pandemic waves are presented in Table 1. The median age was 61 years old (IQR 44, 73), ranging between 18 and 102. The median age increased from 54 years (Wuhan) to 70 years (Omicron). Overall, there was a slightly higher prevalence of female patients.

Cardiovascular diseases were the most prevalent in the Delta wave (64.11%) and the least prevalent in the Wuhan wave (43.01%). Obesity was the highest in the Alpha wave (44.52%) and the lowest in the Omicron wave (18.96%). Diabetes and neurological conditions showed increasing trends, with the highest percentages in the Delta wave. Pulmonary diseases, cancer, and renal and hepatic conditions were the most prevalent in the Omicron wave.

During the Wuhan wave, the majority of patients were hospitalized before the introduction of the vaccination (89.35%). There was an increasing trend in vaccination between the Alpha and Omicron waves.

Severe and critical forms of COVID-19 were the most prevalent in the Delta wave (30.1% severe, 34.37% critical), while asymptomatic and mild cases were more common in the Wuhan wave.

Hydroxychloroquine and lopinavir/ritonavir usage was predominant in the Wuhan wave, while remdesivir and favipiravir were more used in the later waves, especially Delta. The use of newer treatments like Molnupiravir and Casirivimab/Imdevimab was exclusive to the Omicron wave. Corticotherapy and Anakinra showed higher usage rates in the Alpha and Delta waves.

The median hospitalization time was the longest for the Delta wave (10 days). The ICU stay and in-hospital mortality rates were the highest in the Alpha wave (15.48%) and Delta wave (15.05%). Mortality among patients staying in the ICU was the highest in the Delta wave (55.29%), followed by the Omicron wave (47.53%).

Acute pulmonary and non-pulmonary complications were associated with the COVID-19 clinical picture, and a comparison between waves is presented in Table 2. The group of other thrombotic events included different venous thromboses, while the group of other hemorrhagic manifestations included spontaneous atraumatic muscular hematoma.

Pneumothorax/pneumomediastinum (PNX/PNMD) and pulmonary embolisms (PE) were the most common in Delta wave, with incidence rates of 2.97% and 3.4%, respectively. AKI, stroke, GI bleeding, and other thrombotic and hemorrhagic events had the highest incidence rates during the Omicron wave (11.04%, 2.08%, 1.36%, 2.04%, and 3.3%). Liver injury was the most frequent during the Delta wave (34.73%). The highest mortality was observed in patients with pneumothorax/mediastinum (odds ratio of 25.1), followed by acute kidney injury, gastrointestinal bleeding, ischemic/hemorrhagic stroke, and other thromboembotic events (odds ratios of 14.71, 12.64, 12.32, and 10.32).

A comparison between COVID-19 pulmonary and extrapulmonary complications in the ICU and patients hospitalized in the infectious diseases ward is presented in Table 3. All complications were significantly more frequently present in the ICU-hospitalized patients. The highest odds of complications for those hospitalized in the ICU were observed for PNX/PNMD (61.31), followed by ischemic/hemorrhagic stroke, (11.38), while high odds ratios (above 7) were found for PE, MI, GI bleeding, other hemorrhagic and thromboembolic events, and AKI.

**Figure 2:**
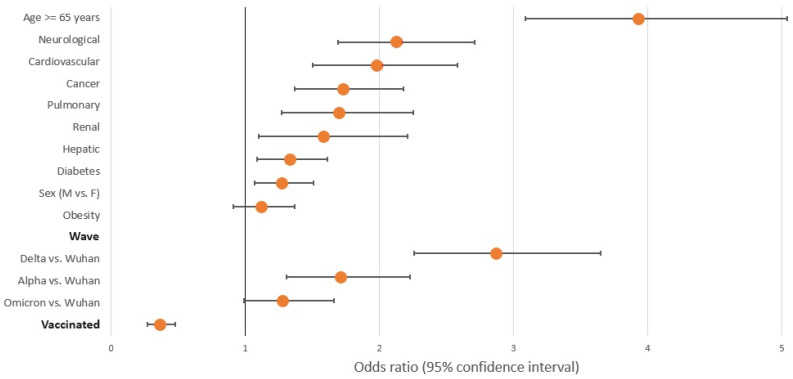
Multiple logistic regression model predicting death.

**Figure 3:**
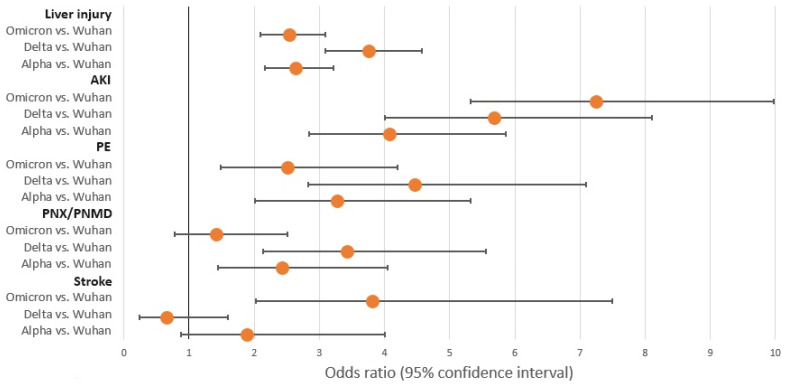
Multiple logistic regression models predicting liver injury, AKI, PE, PNX/PNMD, and stroke, showing only the waves in relation with complications. PE, pulmonary thromboembolism; PNX/PNMD, pneumothorax/pneumomediastinum; AKI, acute kidney injury.

Multiple logistic regression models predicting death and the most frequent complications (liver injury, AKI, PE, PNX/PNMD, and stroke) were built in order to investigate the possible associated factors. The results are presented in Table 4 and Table 5 and in Figure 2 and Figure 3. Mortality was linked to advanced age, male gender, and every analyzed comorbidity, with the exception of obesity. The greatest likelihood of death was recorded in the Delta wave, succeeded by the Alpha wave in comparison to the Wuhan wave, with odds ratios of 2.87 and 1.71, respectively. Vaccination was clearly associated with lower odds of death. To assess the robustness of our results, given the substantial sample size, we re-evaluated the confidence intervals using bias-corrected and accelerated (BCa) bootstrapping with 1000 resamples. This approach yielded confidence intervals that were consistent with those derived from the likelihood ratio statistics. Additionally, we employed 10-fold cross-validation, repeated 100 times, which revealed an overall model accuracy of 93.14% (95% Confidence Interval: 93.09%–93.20%).

Our predictive models indicated elevated risks for acute kidney injury, pulmonary embolism, pneumothorax/pneumomediastinum, and stroke in patients over the age of 65.

Liver injury was more common in individuals under 65 and was linked to factors such as obesity, treatment with corticosteroids, anakinra, favipiravir, and remdesivir, with the Delta variant showing the highest odds ratio (OR).

AKI had associations with comorbidities including cardiovascular diseases, obesity, diabetes, renal issues, and treatments like corticosteroids, anakinra, and remdesivir, with the highest OR observed during the Omicron wave.

PE showed a correlation with male patients but not with obesity, cardiovascular diseases, or cancer, and the highest OR was noted in the Delta wave.

PNX/PNMD were linked to pulmonary comorbidities, with the highest OR during the Delta wave.

Stroke was not related to cardiovascular disease, obesity, or diabetes, but was associated with ages above 65, the Omicron wave, and severe/critical COVID-19 infection.

A history of vaccination reduced the risks of all complications assessed, except for stroke.

## 4. Discussion

To our knowledge, this is the first and largest cohort study in Romania conducted in a single center that compares the clinical features and outcomes of hospitalized COVID-19 adult patients during three pandemic years. We analyzed the patients’ clinical data during different waves caused by SARS-CoV-2 VOCs compared to the ancestral Wuhan strain. It is important to characterize the data from a single center in which a standardized approach to COVID-19 patient care had been implemented. The largest number of patients was hospitalized during the Wuhan wave. This might be explained by the national sanitary decision at the beginning of the pandemic to hospitalize all COVID-19 patients in dedicated hospitals, though an important number of patients were asymptomatic. In comparison, only a few asymptomatic patients were hospitalized during the following waves, when the decision for hospitalization was made based on severity and associated diseases. The largest percentage of patients with severe/critical COVID-19 was described during the Delta wave. The Alpha and Delta VOCs were characterized by increased disease severity, while the Omicron VOC was characterized by a reduced disease severity, compared to the Wuhan strain [12]. Though the Omicron VOC was associated with milder forms [12], during the Omicron wave, more than 33% of patients were hospitalized with severe/critical COVID-19.

Younger patients were hospitalized during the Wuhan wave, while during the Delta and Omicron waves, more than 50% of patients were older than 65, as shown in Table 1. A list of medical conditions associated with the risk of severe COVID-19 is available [13]. In our study, a significant increase in comorbidities (cardiovascular diseases, diabetes, neurological, pulmonary diseases, cancer, and hepatic and renal diseases) was identified during the VOCs’ waves compared to the Wuhan wave, while obesity was more associated with the Alpha and Delta waves, and less to the Wuhan and Omicron waves (Table 1). Another study from our center identified higher odds of severe/critical diseases in patients with diabetes, obesity, cardiovascular, pulmonary diseases, neurological diseases, and cancer, with an ORa of 3.23 for severe/critical COVID-19 in unvaccinated patients [14]. Though vaccination started in Romania at the beginning of January 2021, 78.8% of hospitalized patients were still unvaccinated during the Delta wave, while 46.65% were unvaccinated during the Omicron wave (Table 1). The Omicron VOC was characterized by its capacity to escape prior immunization by vaccination or infection with an increased risk of reinfection [12].

### 4.1. Therapy

Therapy was modified during different waves according to the national protocol, which was first released in March 2020 [15] and updated periodically according to international guidelines. Hydroxychloroquine and lopinavir/ritonavir were used mainly during the first wave, and remdesivir was mainly used during the Alpha and Delta waves, while favipiravir was the most used antiviral in our cohort (39.95%), as shown in Table 1. Favipiravir was included in the national protocol in August 2020 and excluded in the last updated version, which was released in June 2023. Molnupiravir, recommended for outpatient non-severe COVID-19 cases [16], was administered in a small percentage, as patients with non-severe COVID-19 were hospitalized for other associated comorbidities. Nirmatrelvir-ritonavir was only available starting from October 2023 in Romania, and was not administered to any of our patients. Casirivimab/indevimab, the monoclonal antibody with antiviral activity, was surprisingly administered exclusively during the Omicron wave; the majority of patients were treated in January 2022, before data on inefficiency were available [16].

Regarding immunomodulatory therapy, we observed that corticotherapy was over-used. Dexamethasone is recommended in severe/critical COVID-19 cases [16], and though there were 2860 severe/critical COVID-19 patients, corticotherapy was administered in 5012 patients, as shown in Table 1. Tocilizumab, the IL-6 antagonist receptor, was statistically significantly more used during the Alpha and Delta waves and was used less during the Omicron wave, in comparison to the Wuhan wave (Table 1). A retrospective study from our center showed that the early initiation of Tocilizumab seems beneficial in patients with moderate to critical COVID-19 infection [17]. Anakinra, the IL1 antagonist receptor, was included in the national protocol in August 2020. By that time, there were ongoing COVID-19 clinical trials with anakinra [18], while the European Medicine Agency recommended its approval for COVID-19 in December 2021 [19]. A higher percentage of patients were treated with Anakinra (10.52%) compared to Tocilizumab (4.54%); Tocilizumab was the most used during the Alpha wave, while Anakinra was mostly used during the Delta wave (Table 1).

### 4.2. Duration of Hospitalization

The duration of hospitalization is an important indicator of disease severity. During the Wuhan wave, the median seven days of hospitalization is explained not by COVID-19 severity but by the hospitalization of asymptomatic patients, which was mandatory in Romania until 23 June 2020, with two consecutive negative RT-PCR SARS-CoV-2 tests required before discharge. In one previous study, the median time since SARS-CoV-2 PCR negativity was 15 days in asymptomatic/mild cases [20]. The longest hospitalization was associated with the Delta wave (median 10 days), explained by the high percentage of severe/critical COVID-19 cases (30.1% and 34.37%, respectively).

### 4.3. Intensive Care Need

A similar percentage of patients hospitalized in the ICU was described during the Alpha and Delta waves, as presented in Table 1, but the real need during the Delta wave was underestimated due to limited ICU capacity. The percentages of patients hospitalized in the ICU during the Omicron and Wuhan waved were almost similar. Still, the patients were older and associated with more comorbidities (except obesity) during the Omicron wave in comparison to the Wuhan wave.

### 4.4. Outcome

The in-hospital mortality rate was 6.74%, with the highest rate being associated with the Delta (13.6%) wave and the lowest with the Wuhan wave (3.86%) (Table 1). This might be explained by the increased severity of the Delta VOC [12], reduced social distancing measures, and the low uptake of vaccination in the Romanian population in comparison to other European countries [8]. At the end of October 2021, during the Delta wave, Romania ranked in the first position globally in daily COVID-19 deaths/million people [6]. Though a lower mortality was associated with Omicron (6.83%), due to the long period of circulation of the Omicron VOC and an increased number of patients being hospitalized, we may notice that the number of deaths during the Omicron wave (151 patients) is quite close to that associated with the most severe wave, the Delta VOC (188 patients).

### 4.5. Complications of COVID-19

#### 4.5.1. Pulmonary Complications

##### Pulmonary Embolism

A recent review showed that PE incidence was higher in ICU-admitted patients than in overall hospitalized patients [21], similar to our results. The common risk factors for thromboembolic events (cancer, obesity, older age, and history of venous thromboembolism) were not evident in the COVID-19 patients [21]. In our multivariate analysis, cancer, obesity, and cardiovascular comorbidities were not associated with an increased risk of PE, while ages older than 65 years and the male gender were, as shown in Table 5.

A study evaluating the differences in coagulation between hospitalized patients with different VOCs showed that severe coagulation abnormalities may be less likely in Omicron than in patients infected with the Alpha and Delta variants [22]. Similar results were found in our study for PE (Table 1). On the other hand, other thrombotic events (different venous thrombosis) were described with higher incidences during the Omicron wave; the small number of patients with other thrombotic events might explain the differences in the results.

##### Pneumothorax/Pneumomediastinum

Severe lung damage, secondary to a COVID-19 inflammatory response and barotrauma triggered by positive pressure ventilation, may lead to lung injury. A recent review showed that mortality among COVID-19 patients was higher among those who developed PNX/PNMD [23]. Similarly, in our study, as presented in Table 2, the in-hospital mortality rate in patients that developed barotrauma exceeded ten times the overall mortality rate (61.42%; OR = 25.1). AlGhamdi et al. found that the male gender, hypertension, diabetes, endotracheal intubation, and mechanical ventilation were risk factors for PNX [24]. Our multivariate analyses found the following risk factors for PNX/PNMD: age over 65 years, pulmonary comorbidities, and infection during the Alpha and Delta waves (Table 5).

#### 4.5.2. Extrapulmonary Manifestations

##### Liver Injury

Liver injury was the most frequently described extrapulmonary manifestation (17.28%) in our study group, as presented in Table 1. The data on liver injury in COVID-19 patients are heterogeneous [1]—a systematic review indicated mild liver function in every fourth to fifth patient [25]. In our analyses, liver injury was associated with obesity and therapy (corticosteroids, anakinra, favipiravir, and remdesivir), while the highest OR was associated with the Delta wave, as presented in Table 5.

##### Acute Kidney Injury

AKI is now known to be a common COVID-19 complication, but it is unclear whether SARS-CoV-2 affects the kidneys directly or indirectly, similar to other infections [26]. AKI was identified in 5.95% of our patients (Table 2), but the incidence in ICU patients was 23.18%, as presented in Table 3. A recent review reported an AKI incidence of 28–34% in inpatients and 46–77% in ICU patients [26], but the incidence of severe AKI in ICU patients appeared to have declined over time [27]. An explanation of a reduced incidence over time might be the introduction of effective therapies and vaccinations for COVID-19. Surprisingly, our study recorded the highest incidence during the Omicron wave (11.04%), as shown in Table 2. Several risk factors for AKI in COVID-19 patients have been reported (male gender, older age, and pre-existing comorbidities including diabetes, obesity, and chronic kidney disease [28]), similar to the results obtained in our regression model (as presented in Table 5), except for the male gender.

Concerns regarding the remdesivir kidney safety profile were signaled from the initial trials. A pharmacovigilance study reported that AKI was associated with remdesivir use in COVID-19 patients [29]. On the other hand, a recent review suggested that remdesivir treatment was not associated with a significant change in the risk of AKI [30]. In our regression model, an OR of 1.3 (1.04–1.6) was found for AKI in the case of remdesivir use (*p* < 0.05) (Table 5).

##### Ischemic/Hemorrhagic Stroke

In a retrospective cohort of COVID-19 patients published at the beginning of the pandemic, two-thirds developed neurological manifestations during the course of the disease [31]. A review published in 2022 of COVID-19 neurological manifestations identified stroke risk in the range of 0.5–1.6%; ischemic stroke predominated [32]. Interestingly, the highest incidence was found in our study during the Omicron wave (2.08%), as shown in Table 2. Most patients with cerebrovascular complications have traditional risk factors, but a minority do not, suggesting potentially important roles of cytokine storm and the hypercoagulable state [33]. Similarly, in our regression model, presented in Table 5, comorbidities like cardiovascular disease, obesity, and diabetes were not associated with stroke, but ages over 65 and severe/critical COVID-19 cases were associated, supporting the role of cytokine storm in pathogenesis. However, as discussed, even if coagulopathy was more described during the Delta wave, hospitalization associated with stroke was more frequent during the Omicron wave. A high in-hospital mortality rate (52.7%) in patients with intracerebral hemorrhage and COVID-19 was reported in a review, with no data available for a longer follow–up [34]. The high in-hospital mortality rate in stroke (45.68%) in our study (Table 2) reinforces that this is a life-threatening COVID-19 complication.

##### Myocardial Infarction (MI)

A hyperinflammatory state with plaque rupture events, as well as the upregulation of procoagulants and platelet activation, are proposed mechanisms for MI in COVID-19. MI is described more frequently in COVID-19 patients with preexisting cardiovascular disease [4]. At the beginning of the pandemic, high prevalences of MI, ranging from 5% to 38%, were reported [35] with increased mortality rates [36]. Updated data from the North American COVID-19 STEMI registry showed that, after COVID-19 vaccination started, there was a reduction in in-hospital mortality, with a trend towards prepandemic evolution in patients with COVID-19 and STEMI [37].

In our study, all VOCs were associated with a higher prevalence of MI in comparison to the Wuhan wave, while the overall in-hospital mortality rate was 29.17%, as presented in Table 2.

##### Gastrointestinal (GI) Bleeding

A recent review reported a range of GI bleeding from 1.1% to 13% in COVID-19 patients [38], but it is rarely the initial manifestation of COVID-19 [39]. The host immune response, including hyperinflammation, multiorgan viral dysfunction, coagulopathy, and adverse reactions to medication (corticosteroids and anticoagulants), may be involved in pathogenesis.

A large COVID-19 cohort, mostly from Egypt, reported a high rate of GI bleeding from hematemesis in 9%, melena in 5.3%, hematochezia in 0.6%, and fecal-occult positive stools in 5% [40]. Contrariwise, a systematic review of 12 studies reported a prevalence of GI bleeding of only 0.06% [41]. A European cohort of 4128 COVID-19 subjects noted a 1.8% prevalence of GI bleeding [42]. In a Polish one-center study, GI was most frequently described in patients hospitalized during the first pandemic wave [43]. GI bleeding was described in 60 patients from our study (0.66%), with the highest incidence during the Omicron wave (Table 2), and an OR of 9.28 in ICU-hospitalized patients (Table 3). Similarly, other hemorrhagic manifestations (atraumatic muscular hematoma) were mostly described during the Omicron wave (3.3%), as shown in Table 2.

### 4.6. Limitations and Strengths

We separated the study interval into waves based on national data on the predominance of different VOCs, but the co-circulation of different VOCs existed, and no individual VOC identification was available, limiting the absolute association of patients to the different VOCs. As the data were collected retrospectively, the COVID-19 complications were recorded as found in the patients’ electronic files and according to the clinicians’ discharge diagnoses, and no interpretation of clinical and paraclinical data was performed by the study investigators.

Nonetheless, our study features notable strengths, notably its substantial sample size, extensive timeframe, and the unique focus on COVID-19 clinical manifestations and outcomes across various pandemic waves within a dedicated COVID-19 center. This research is highly relevant in the context of emerging variants, as it offers invaluable real-world insights into disease severity, both pulmonary and extrapulmonary manifestations, the influence of comorbidities, treatment strategies, vaccination statuses, and patient outcomes, all gathered over the course of three challenging pandemic years.

## 5. Conclusions

In conclusion, our study highlights the changing evolution of inpatient characteristics and outcomes associated with different waves. Pulmonary and extrapulmonary complications were present more frequently in ICU-hospitalized patients and with differences between waves. The highest in-hospital mortality rate was described in patients presenting PNX/PNMD and during the Delta wave. PE and liver injury had the highest incidence rates during the Delta wave. AKI, stroke, GI bleeding, and other hemorrhagic and thrombotic events had the highest incidence rates during the Omicron wave. All of the evaluated risk factors were significantly associated with death, except obesity and the Omicron wave. Our data indicate that for all four waves, COVID-19 was a severe disease in hospitalized patients with a high risk of poor outcomes. Our study underlines the changing nature of COVID-19 and acknowledges the impact of viral mutations on disease outcomes.

## Figures and Tables

**Table 1:** Patients’ demographics, comorbidities, vaccination statuses, COVID-19 severity forms, therapies, and outcomes.

Characteristic	Total (*n* = 9049)	Wuhan (*n* = 4197) 46.38%	Alpha (*n* = 1260) 13.92%	Delta (*n* = 1382) 15.27%	Omicron (*n* = 2210) 15.27%	*p*-Value
**Demographic data**						
Age (years), median (IQR)	61 (44, 73)	54.0 (40.0–67.0)	63.0 (51.0–72.0)	64.5 (51.0–75)	70.0 (49.0–80.0)	<0.001
Age ≥ 65 years, *n* (%)	3906 (43.16)	1264 (30.12)	577 (45.79)	691 (50)	1374 (62.17)	<0.001
Age ≥ 70 years, *n* (%)	2951 (32.61)	867 (20.66)	421 (33.41)	519 (37.55)	1144 (51.76)	<0.001
Sex (female), *n* (%)	4935 (54.54)	2227 (53.06)	626 (49.68)	761 (55.07)	1321 (59.77)	<0.001
**Comorbidities**						
Cardiovascular, *n* (%)	4730 (52.27)	1805 (43.01)	749 (59.44)	886 (64.11)	1290 (58.37)	<0.001
Obesity, *n* (%)	2335 (25.8)	878 (20.92)	561 (44.52)	477 (34.52)	419 (18.96)	<0.001
Diabetes, *n* (%)	1720 (19.01)	665 (15.84)	277 (21.98)	330 (23.88)	448 (20.27)	<0.001
Neurological, *n* (%)	915 (10.11)	254 (6.05)	119 (9.44)	172 (12.45)	370 (16.74)	<0.001
Pulmonary, (*n* (%)	892 (9.86)	264 (6.29)	129 (10.24)	191 (13.82)	308 (13.94)	<0.001
Cancer, *n* (%)	654 (7.23)	229 (5.46)	69 (5.48)	102 (7.38)	254 (11.49)	<0.001
Renal, *n* (%)	484 (5.35)	159 (3.79)	55 (4.37)	97 (7.02)	173 (7.83)	<0.001
Hepatic, (*n* (%)	429 (4.74)	154 (3.67)	50 (3.97)	89 (6.44)	136 (6.15)	<0.001
Vaccination status, *n* (%)						<0.001
0/1	3813 (42.14)	446 (10.63)	1247 (98.97)	1089 (78.8)	1031 (46.65)	
2	935 (10.33)	1 (0.02)	13 (1.03)	272 (19.68)	649 (29.37)	
3	548 (6.06)	0 (0)	0 (0)	21 (1.52)	527 (23.85)	
4	3 (0.03)	0 (0)	0 (0)	0 (0)	3 (0.14)	
Prevaccination	3750 (41.44)	3750 (89.35)	0 (0)	0 (0)	0 (0)	
Severity form, *n* (%)						<0.001
Asymptomatic	549 (6.07)	524 (12.49)	7 (0.56)	1 (0.07)	17 (0.77)	
Mild	1811 (20.01)	989 (23.56)	103 (8.17)	122 (8.83)	597 (27.01)	
Medium	3829 (42.31)	1996 (47.56)	628 (49.84)	368 (26.63)	837 (37.87)	
Severe	1881 (20.79)	548 (13.06)	437 (34.68)	416 (30.1)	480 (21.72)	
Critical	979 (10.82)	140 (3.34)	85 (6.75)	475 (34.37)	279 (12.62)	
**Therapy**						
Hydroxychloroquine, *n* (%)	1736 (19.18)	1680 (40.03)	43 (3.41)	13 (0.94)	0 (0)	<0.001
Lopinavir/ritonavir, *n* (%)	653 (7.22)	653 (15.56)	0 (0)	0 (0)	0 (0)	<0.001
Remdesivir, *n* (%)	1468 (16.22)	360 (8.58)	345 (27.38)	417 (30.17)	346 (15.66)	<0.001
Favipiravir, *n* (%)	3615 (39.95)	433 (10.32)	957 (75.95)	1105 (79.96)	1120 (50.68)	<0.001
Molnupiravir, *n* (%)	177 (1.95)	0 (0)	0 (0)	0 (0)	177 (8.01)	<0.001
Casirivimab/imdevimab, *n* (%)	152 (1.67)	0 (0)	0 (0)	0 (0)	152 (6.88)	<0.001
Corticotherapy, *n* (%)	5012(55.39)	1734 (41.32)	1079 (85.63)	1130 (81.77)	1069 (48.37)	<0.001
Anakinra, *n* (%)	952(10.52)	152 (3.62)	279 (22.14)	392 (28.36)	129 (5.84)	<0.001
Tocilizumab, *n* (%)	411(4.54)	153 (3.65)	138 (10.95)	94 (6.8)	26 (1.18)	<0.001
Anticoagulant, *n* (%)	7022 (77.6)	2850 (67.91)	1167 (92.62)	1300 (94.07)	1705 (77.15)	<0.001
**Outcome**						
Hospitalization time (days), median (IQR)	7 (4, 12)	7 (4–12)	8 (5–12)	10 (6–15)	7 (4–12)	<0.001
ICU stay, *n* (%)	1018 (11.25)	392 (9.34)	195 (15.48)	208 (15.05)	223 (10.09)	<0.001
In-hospital mortality, *n* (%)	610 (6.74)	162 (3.86)	109 (8.65)	188 (13.6)	151 (6.83)	<0.001
Mortality in patients staying in ICU, *n* (%)	432 (42.44)	134 (34.18)	77 (39.49)	115 (55.29)	106 (47.53)	<0.001

IQR, interquartile range; 0/1, 0 means unvaccinated, 1 means incomplete vaccination (one dose in vaccination with two doses in primary vaccination); 2, complete primary vaccination; 3, first booster vaccination; 4, second booster vaccination; ICU, intensive care unit. Prevaccination indicates the vaccination status when a patient did not receive any vaccine dose, because at the time of hospitalization, there were no vaccines available on the market.

**Table 2:** Incidence rates of pulmonary and non-pulmonary complications and outcomes (death).

Complications	All	Wuhan (*n* = 4197)	Alpha (*n* = 1260)	Delta (*n* = 1382)	Omicron (*n* = 2210)	*p*-Value	Died	OR (95% CI)	*p*-Value
**Pulmonary**									
PNX/PNMD, *n* (%)	127 (1.4)	34 (0.81)	28 (2.22)	41 (2.97)	24 (1.09)	<0.001	78 (61.42)	25.1 (17.38–36.27)	<0.001
PE, *n* (%)	153 (1.69)	34 (0.81)	36 (2.86)	47 (3.4)	36 (1.63)	<0.001	43 (28.1)	5.74 (4–8.25)	<0.001
**Extrapulmonary**									
Other thrombotic events, *n* (%)	82 (0.91)	7 (0.17)	10 (0.79)	20 (1.45)	45 (2.04)	<0.001	43 (28.1)	10.32 (6.6–16.14)	<0.001
Liver injury, *n* (%)	1564 (17.28)	337 (8.03)	360 (28.57)	480 (34.73)	387 (17.51)	<0.001	114 (7.29)	1.11 (0.9–1.37)	0.342
AKI, *n* (%)	538 (5.95)	66 (1.57)	92 (7.3)	136 (9.84)	244 (11.04)	<0.001	222 (41.26)	14.71 (12.05–17.96)	<0.001
Ischemic/hemorrhagic stroke, *n* (%)	85 (0.94)	18 (0.43)	14 (1.11)	7 (0.51)	46 (2.08)	<0.001	37 (45.68)	12.32 (7.89–19.23)	<0.001
MI, *n* (%)	24 (0.27)	5 (0.12)	6 (0.48)	3 (0.22)	10 (0.45)	0.023	7 (29.17)	5.75 (2.38–13.92)	<0.001
Gastrointestinal bleeding, *n* (%)	60 (0.66)	18 (0.43)	2 (0.16)	10 (0.72)	30 (1.36)	<0.001	28 (46.67)	12.64 (7.56–21.13)	<0.001
Other hemorrhagic events, *n* (%)	104 (1.15)	11 (0.26)	12 (0.95)	8 (0.58)	73 (3.3)	<0.001	27 (25.96)	5.03 (3.22–7.86)	<0.001

PNX/PNMD, pneumothorax/pneumomediastinum; PE, pulmonary thromboembolism; AKI, acute kidney injury; MI, myocardial infarction; OR, odds ratio of death; CI, confidence interval.

**Table 3:** COVID-19 complications in patients hospitalized in ICU and infectious diseases ward.

Complications	ICU (*n* = 1018)	Infectious Diseases Ward (*n* = 8031)	OR (95% CI)	*p*-Value
PNX/PNMD, *n* (%)	111 (10.9)	16 (0.2)	61.31 (36.14–104.01)	<0.001
PE, *n* (%)	81 (7.96)	72 (0.9)	9.56 (6.91–13.22)	<0.001
Other thromboembolic events, *n* (%)	39 (3.83)	43 (0.54)	7.4 (4.77–11.47)	<0.001
Liver injury, *n* (%)	236 (23.18)	1328 (16.54)	1.52 (1.3–1.78)	<0.001
AKI, *n* (%)	240 (23.58)	298 (3.71)	8.01 (6.65–9.63)	<0.001
Stroke ischemic/hemorrhagic *n* (%)	47 (4.62)	34 (0.42)	11.38 (7.29–17.79)	<0.001
MI, *n* (%)	13 (1.28)	11 (0.14)	9.43 (4.21–21.11)	<0.001
Gastrointestinal bleeding, *n* (%)	32 (3.14)	28 (0.35)	9.28 (5.56–15.47)	<0.001
Other hemorrhagic events, *n* (%)	53 (5.21)	51 (0.64)	8.59 (5.82–12.7)	<0.001

ICU, intensive care unit; OR, odds ratio; CI, confidence interval; PNX/PNMD, pneumothorax/pneumomediastinum; PE, pulmonary thromboembolism; AKI, acute kidney injury; MI, myocardial infarction.

**Table 4:** Multiple logistic regression model predicting death.

Characteristics	OR-Adjusted	(95% CI)	(95% BCa CI)	*p*
Age ≥ 65 years	3.93	(3.09–5.04)	(3.1–5.16)	<0.001
Neurological	2.42	(1.95–3)	(1.93–3.03)	<0.001
Cardiovascular	2.13	(1.69–2.71)	(1.68–2.77)	<0.001
Cancer	1.98	(1.5–2.58)	(1.48–2.64)	<0.001
Pulmonary	1.73	(1.37–2.18)	(1.35–2.23)	<0.001
Renal	1.7	(1.27–2.25)	(1.27–2.32)	<0.001
Hepatic	1.58	(1.1–2.21)	(1.05–2.25)	0.011
Diabetes	1.33	(1.09–1.61)	(1.08–1.62)	0.004
Sex (M vs. F)	1.27	(1.07–1.51)	(1.06–1.51)	0.008
Obesity	1.12	(0.91–1.37)	(0.9–1.39)	0.278
Wave				
Delta vs. Wuhan	2.87	(2.26–3.65)	(2.23–3.63)	<0.001
Alpha vs. Wuhan	1.71	(1.31–2.23)	(1.32–2.2)	<0.001
Omicron vs. Wuhan	1.28	(0.99–1.66)	(0.98–1.67)	0.063
Vaccinated	0.36	(0.27–0.48)	(0.26–0.48)	<0.001

OR, odds ratio; CI, confidence interval; BCa CI, bias-corrected and accelerated bootstrapped confidence intervals with 1000 samples.

**Table 5:** Multiple logistic regression models predicting liver injury, AKI, PE, PNX/PNMD, and stroke.

	Predicted Complications														
	Liver Injury				AKI		PE			PNX/PNMD			Stroke		
Independent Predictors	OR	95% CI	*p*	OR	95% CI	*p*	OR	95% CI	*p*	OR	(95% CI)	*p*	OR	95% CI	*p*
Age ≥ 65 years	0.81	0.71–0.91	<0.001	3.47	2.67–4.54	<0.001	2.2	1.49–3.28	<0.001	1.77	(1.16–2.74)	0.009	2.24	1.2–4.43	0.015
Sex (M vs. F)	1.12	1–1.26	0.057	1.18	0.97–1.43	0.091	1.56	1.13–2.17	0.007	1.13	(0.79–1.61)	0.497	1.53	0.97–2.42	0.068
Cardiovascular				1.75	1.36–2.26	<0.001	1.04	0.71–1.53	0.859	1.2	(0.79–1.85)	0.401	1.4	0.79–2.61	0.264
Obesity	1.28	1.13–1.46	<0.001	1.27	1.02–1.57	0.028	0.86	0.58–1.24	0.425	0.92	(0.6–1.38)	0.698	0.73	0.4–1.27	0.288
Diabetes	0.85	0.73–0.99	0.032	1.24	1–1.52	0.044				1.09	(0.7–1.65)	0.688	0.77	0.43–1.29	0.339
Pulmonary										2.54	(1.65–3.82)	<0.001			
Cancer	0.83	0.66–1.04	0.113	1.32	0.98–1.76	0.063	0.82	0.41–1.47	0.535	1.53	(0.82–2.63)	0.15			
Renal				1.83	1.38–2.42	<0.001									
Hepatic	1.15	0.89–1.47	0.291												
Corticotherapy	1.84	1.59–2.13	<0.001	2.38	1.82–3.12	<0.001									
Tocilizumab	0.87	0.67–1.12	0.289	1.01	0.67–1.46	0.979									
Anakinra	1.53	1.3–1.8	<0.001	1.94	1.53–2.46	<0.001									
Favipiravir	1.35	1.17–1.55	<0.001	0.41	0.34–0.51	<0.001									
Remdesivir	1.21	1.04–1.4	0.014	1.3	1.04–1.6	0.018									
Wave															
Alpha vs. Wuhan	2.63	2.16–3.21	<0.001	4.07	2.84–5.86	<0.001	3.27	2.01–5.32	<0.001	2.42	(1.44–4.05)	<0.001	1.89	0.88–4.01	0.097
Delta vs. Wuhan	3.76	3.09–4.58	<0.001	5.68	4–8.11	<0.001	4.46	2.83–7.1	<0.001	3.43	(2.13–5.56)	<0.001	0.65	0.24–1.59	0.358
Omicron vs. Wuhan	2.55	2.1–3.09	<0.001	7.24	5.32–9.97	<0.001	2.51	1.49–4.2	<0.001	1.42	(0.78–2.51)	0.238	3.81	2.02–7.5	<0.001
Vaccinated	0.79	0.66–0.94	0.008	0.72	0.55–0.92	0.01	0.33	0.18–0.59	<0.001	0.36	(0.18–0.69)	0.003	0.54	0.28–1	0.058
Severe or critical													5.1	2.93–9.32	<0.001

Five multiple logistic regression models are presented, predicting COVID-19 complications. For each model, odds ratios are presented only for the included independent predictors. PE, pulmonary thromboembolism; PNX/PNMD, pneumothorax/pneumomediastinum; AKI, acute kidney injury; OR, odds ratio; CI, confidence interval.

## Data Availability

Data are available upon request due to restrictions, e.g., privacy or ethical restrictions.
